# Sultan’s Score: A Novel Predictive Score to Predict Complete Response Following Drug-Eluting Bead Chemoembolization

**DOI:** 10.7759/cureus.76822

**Published:** 2025-01-02

**Authors:** Sultan R Alharbi

**Affiliations:** 1 Radiology and Medical Imaging, King Saud University, Riyadh, SAU

**Keywords:** chemoembolization, complete response, drug-eluting beads, hepatocellular carcinoma, partial response, prediction score, prognosis

## Abstract

Background: Transcatheter arterial chemoembolization (TACE) is a well-known standard treatment for hepatocellular carcinoma (HCC); however, the effectiveness of this treatment can vary among patients.

Objectives: This study aimed to develop a novel imaging-based prediction score (Sultan’s score) to predict complete response after treatment.

Methods: From January 2015 to 2021, 41 patients with solitary HCC, admitted at King Saud University Medical City, Riyadh, Saudi Arabia, were treated with drug-eluting TACE (DEBTACE). Clinical data, tumor details, treatment specifics, and outcomes were gathered retrospectively. Sultan's score incorporates five imaging-based elements, namely, well-defined tumor borders, presence of tumor capsule, tumor size, arterial hyper enhancement, and hypertrophic arterial feeder. The cut-off value of Sultan's score was determined by calculating the maximum Youden index using the receiver operating characteristic curve to accurately predict complete treatment response to DEBTACE.

Results: Following two DEBTACE sessions, 28 (68.3%) and 13 (31.7%) patients showed complete and partial responses, respectively. The mean ± standard deviation and median (interquartile range) of the Sultan’s score in patients with a complete treatment response were 3.93 ± 0.72 and 4 (4-4), and the corresponding values in patients with a partial response were 2.77 (0.93) and 3 (2-3.5), respectively. A cut-off value of 3.5 for the Sultan’s score had a sensitivity and specificity of 78.6% and 76.9%, respectively, in predicting a complete treatment response. The area under the curve was 0.827 (95% confidence interval: 0.688-0.966).

Conclusions: We developed a novel imaging-based scoring system (Sultan’s score) for predicting complete response in patients with HCC following DEBTACE.

## Introduction

Transcatheter arterial chemoembolization (TACE) is the most commonly used primary treatment modality for unresectable hepatocellular carcinoma (HCC) [1-3]. However, the response to TACE greatly varies and is dependent on multiple factors, including tumor burden, tumor biology, and embolization procedure technique [4]. Given the heterogeneity of patients with HCC, including differences in tumor size, vascularization, treatment history, and background liver disease and function, determining the treatment efficacy of TACE is difficult [5]. Several scoring systems have been developed to identify patients likely to benefit from TACE. However, none of these models has been firmly recommended by existing guidelines. Therefore, an easy-to-use prediction scoring system for guiding individualized management of patients with HCC is needed [6]. It is important to identify predictors of complete response after TACE [7].

Numerous studies have confirmed a correlation between imaging biomarkers and the TACE response [8,9]; for example, well-defined smooth margins, smaller size, encapsulated tumor, and hypervascularity of HCC nodules are associated with better TACE response. Infiltrative HCC with ill-defined margin, lack of capsule, hypovascular, and larger HCC are associated with poor responses to TACE [10,11].

This study aimed to formulate a simple score based on imaging features, including HCC size, margin, capsule, enhancement, and hypertrophic arterial supply, for predicting the response to TACE.

This article was previously presented as a meeting abstract at the European Conference on Interventional Oncology (ECIO) on April 28, 2024.

## Materials and methods

Study design

We retrospectively collected data from consecutive patients who underwent DEBTACE treatment for HCC between January 2015 and January 2021 at King Saud University Medical City, a tertiary university hospital in Riyadh, Saudi Arabia. While this design may introduce selection bias, we ensured data completeness by including all consecutive patients and performing rigorous data cross-checking. The inclusion criteria were as follows: age >18 years, Child-Pugh score A-B, solitary HCC, and no vascular invasion or extrahepatic metastasis. The exclusion criteria were multiple HCCs, vascular invasion, prior or adjuvant HCC therapy, and incomplete data. The study was conducted in accordance with the Declaration of Helsinki and reviewed and approved by King Saud University Affiliated University Hospital Institutional Review Board (approval no. E-21-6418).

Protocol for DEBTACE

Following local anesthetic infiltration of the subcutaneous tissue, the common femoral artery was punctured using the Seldinger technique under aseptic conditions and ultrasound guidance, followed by placement of a 5-French vascular sheath. We used a 5-French angled catheter and wire to select the celiac and mesenteric arteries. Angiography of the celiac trunk and mesenteric arteries was performed to delineate the arteries feeding the HCC tumor. A microcatheter and microwire were used for super selection of the feeding arteries as distally as possible. Subsequently, one vial of 100-300 microns of drug-eluting microspheres loaded with 75 mg doxorubicin was slowly injected into the feeding arteries until there was almost complete stasis. Large tumors required two vials of drug-eluting beads loaded with 150 mg of doxorubicin. DEBTACE treatment followed standard protocol [12].

New prediction scoring system

**Figure 1:**
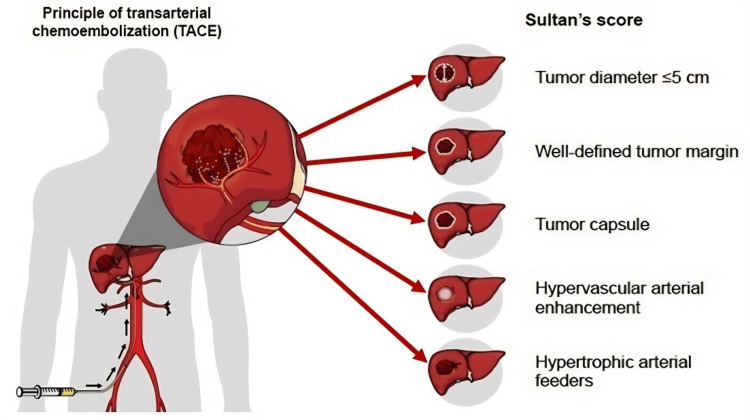
New prediction score "Sultan's Score" Diagram of five elements of the score. (Image created by Sultan Alharbi)

We defined a new scoring system "Sultan’s score" comprising five elements of baseline computed tomography (CT) or magnetic resonance imaging (MRI). The five elements include tumor size ≤5 cm in maximum diameter, presence of well-defined margins of all tumor borders, presence of tumor capsule which appeared as peripheral smooth delayed enhancing rim surrounding the tumor, hypervascular arterial enhancement, and hypertrophic arterial supply. Each element is assigned a score of 1, with the maximum and minimum values being 5 and 0, respectively (Figure 1).

The hypertrophic arterial supply, although not a feature of HCC, is used as guidance for the TACE technique to identify and embolize the arterial supply to HCC in order to achieve a complete response (CR).

Follow-up and response evaluation

All patients underwent contrast-enhanced CT or MRI during follow-up at four to eight weeks after DEBTACE. Response evaluation was performed based on modified response evaluation criteria in solid tumors (mRECIST).

Statistical analysis

Statistical analyses were performed using IBM SPSS Statistics for Windows, Version 25.0 (released 2017, IBM Corp., Armonk, NY). A total of 18 variables were included in the data analysis. Chi-squared and Fisher’s exact tests were used to test the statistical significance of the cross-tabulation between categorical variables. The normality of the data distribution of continuous variables was checked using the Kolmogorov-Smirnov and Shapiro-Wilk tests. Given the non-normal dT distribution of the variables, the Mann-Whitney U test was used for between-group comparisons. The maximum Youden index calculated by subtracting (1-specificity) from the sensitivity, was used to determine the cutoff value on the receiver operating characteristic (ROC) curve for predicting the CR of HCC to DEBTACE. Statistical significance was set at P < 0.05.

## Results

Bassline characteristics of patients

**Figure 2:**
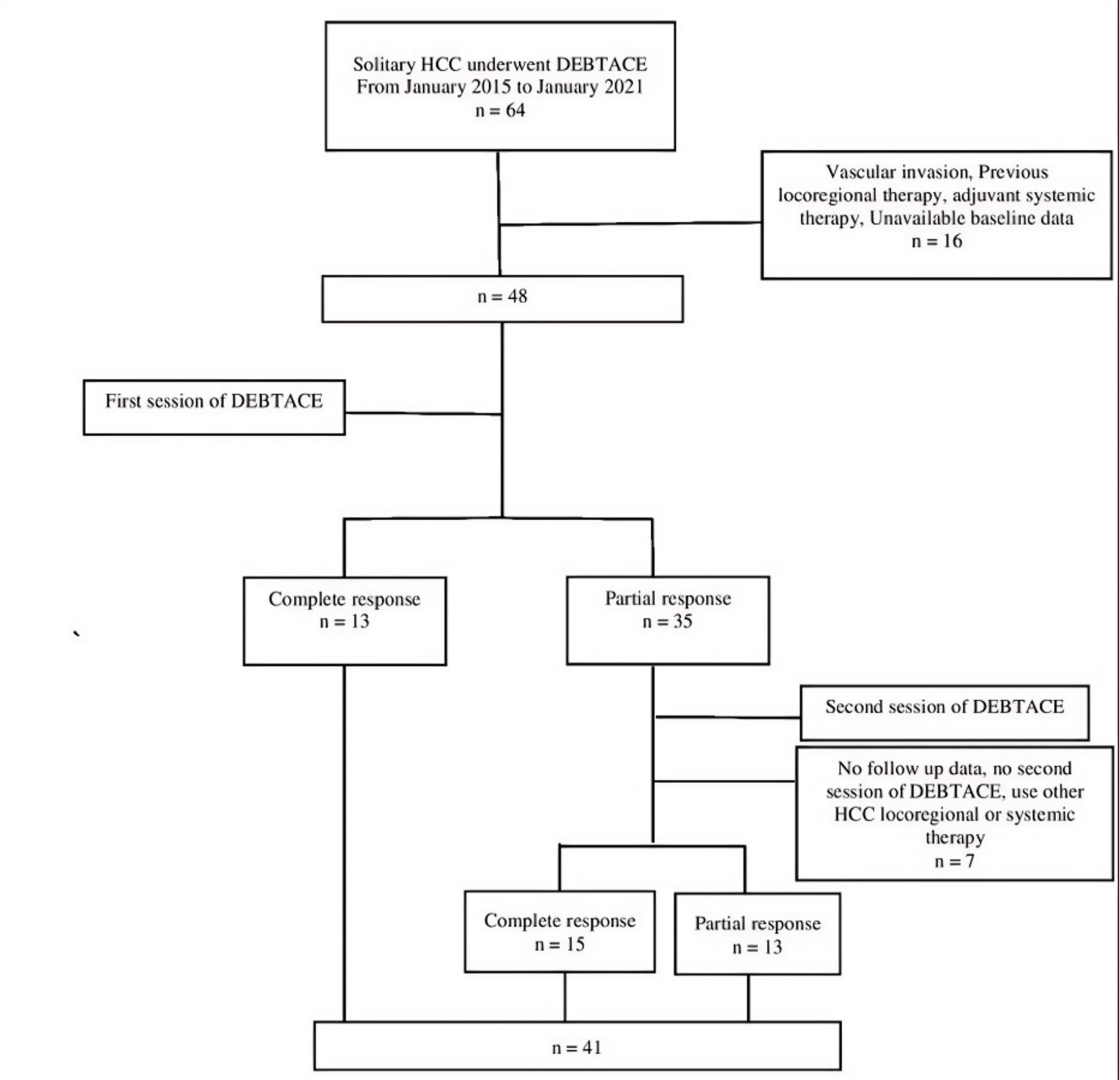
Patient enrollment flow chart

We included 41 patients with solitary HCC who underwent DEBTACE as the primary treatment at a single institution. Figure 2 presents the study flow chart.

The mean ± standard deviation (SD) and median (interquartile range (IQR)) age of the participants were 68.29 ± 12.79 years and 70 (61.50-76.50) years, respectively. The study participants comprised 28 (68.3%) men and 13 (31.7%) women.

Hepatitis C virus was the most common etiology (18 (43.9%)), followed by hepatitis B virus (11 (26.8%)). Moreover, 35 (85.4%) and 6 (14.6%) participants had Child-Pugh scores of A and B, respectively. Alpha-fetoprotein (AFP) levels were normal and elevated in 20 (48.8%) and 21 (51.2%) patients, respectively. These data are presented in Table 1.

**Table 1:** Patients' baseline characteristics AFP, alpha-fetoprotein; AIH, autoimmune hepatitis; HBV, hepatitis B virus; HCV, hepatitis C virus; NASH, nonalcoholic steatohepatitis

Variable	Value
Sex, n (%)	
Female	13 (31.7)
Male	28 (68.3)
Age (years)	70 (61–76)
Etiology, n (%)	
HCV	18 (43.9)
HBV	11 (26.8)
NASH	7 (17.2)
Alcoholic	1 (2.4)
AIH	1 (2.4)
Non-cirrhotic	3 (7.3)
Child–Pugh Score, n (%)	
A	35 (85.4)
B	6 (14.6)
AFP, n (%)	
Normal	20 (48.8)
Raised	21 (51.2)

HCC characteristics

The mean ± SD and median (IQR) size of the HCC lesions were 4.63 ± 2.30 cm and 4.20 (2.80-6.00) cm, respectively. The minimum and maximum sizes were 2 and 10.5 cm, respectively, with a range of 8.5 cm. Most HCC lesions were in segment 8 (n = 14 (34.1%)), followed by segments 5 and 7 (both n = 5 (12.2%)). The details are presented in Table 2.

**Table 2:** Characteristics of hepatocellular carcinoma (HCC) lesions

Characteristics of HCC lesions		Number	Percentage
Location	1	3	7.3
	2 and 3	1	2.4
	2	4	9.8
	4A	3	7.3
	5	5	12.2
	5 and 6	1	2.4
	6	4	9.8
	7 and 8	1	2.4
	7	5	12.2
	8	14	34.2
Vascularity	Hyper vascular	39	95.1
	Hypo vascular	2	4.9
Border	Well defined	37	90.2
	Ill defined	4	9.8
Capsule	Absent	27	65.9
	Present	14	34.1
Hypertrophic arterial supply	Absent	14	34.1
	Present	27	65.9

Sultan’s score

The minimum and maximum Sultan’s scores were 1 and 5, respectively. One (2.4%), five (12.2%), 10 (24.4%), 20 (48.8%), and five (12.2%) patients had Sultan’s scores of 1, 2, 3, 4, and 5, respectively. The mean ± SD and median (IQR) values of the Sultan’s score were 3.56 ± 0.95 and 4 (3-4), respectively.

Response to treatment

Among the 41 patients, 28 (68.3%) showed CR while 13 (31.7%) showed PR. Patients with CR were significantly younger than those with PR (P = 0.031). The lesion size was significantly smaller in patients with a CR than in those with a PR (P = 0.033).

There were significant differences in the Child-Pugh score (P = 0.046), lesion borders (P = 0.050), and capsule (P = 0.015) according to the treatment response. These data are presented in Table 3.

**Table 3:** Association between treatment response and variables. * significant P-value; # Fisher's exact test; NA not available; X2 means chi-square

Variable		Treatment response		X2	P value
		Complete	Partial		
		N (%)	N (%)		
Gender	Female	8 (61.5)	5 (38.5)	0.401	0.527
	Male	20 (71.4)	8 (28.6)		
Child Pugh score	A	26 (74.3)	9 (25.7)	3.967	0.046*
	B	2 (33.3)	4 (66.7)		
AFP	Normal	14 (70.0)	6 (30.0)	0.053	0.819
	Raised	14 (66.7)	7 (33.3)		
Vascularity	Hyper vascular	28 (71.8)	11 (28.2)	NA	0.095^#^
	Hypo vascular	0 (0)	2 (100)		
Borders	Ill defined	1 (25)	3 (75)	3.837	0.050*
	Well defined	27 (73)	10 (27)		
Capsule	Absent	15 (55.6)	12 (44.4)	5.924	0.015*
	Present	13 (92.9)	1 (7.1)		
Hypertrophic arterial supply	Absent	8 (57.1)	6 (42.9)	1.221	0.269
	Present	20 (74.1)	7 (25.9)		
Number of TACE sessions	1	14 (100)	0 (0)	NA	0.001*^#^
	2	14 (51.9)	13 (48.1)		

Association between Sultan’s score and treatment response

All patients with a Sultan’s score of 5 showed a CR, with the proportion of patients with a CR decreasing with a decreasing Sultan’s score; specifically, 85%, 50%, 20%, and 0% of patients had Sultan’s scores of 4, 3, 2, and 1, respectively (P = 0.007; Table 4)

**Table 4:** Association between Sultan’s score and treatment response.

Sultan’s score	Complete treatment response	Partial treatment response	X^2^	P value
	N (%)	N (%)		
1	0 (0)	1 (100)	13.984	0.007*
2	1 (20)	4 (80)		
3	5 (50)	5 (50)		
4	17 (85)	3 (15)		
5	5 (100)	0 (0)		

**Figure 3:**
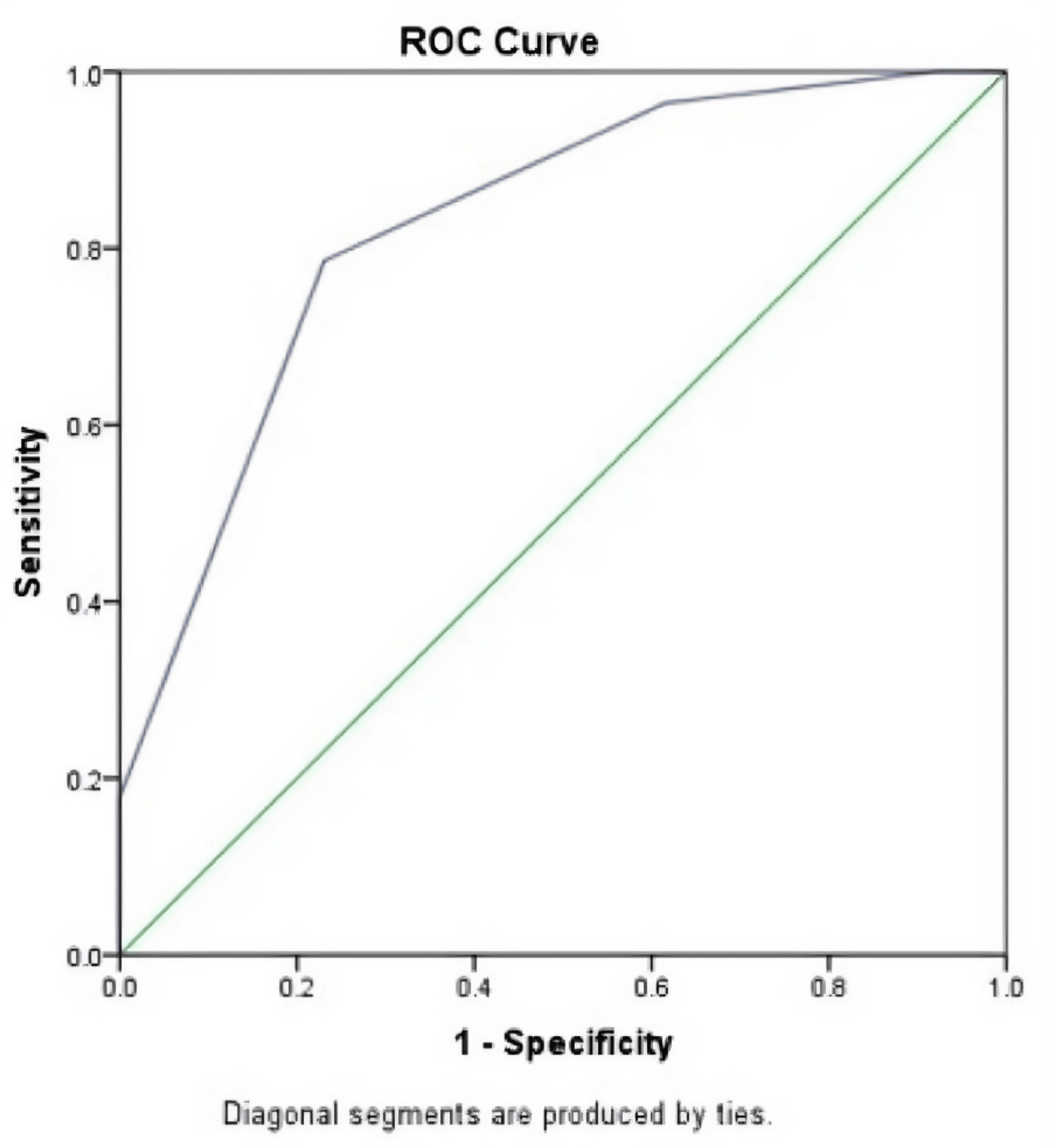
ROC curve analysis of Sultan's score for predicting a complete treatment response. ROC: receiver operating characteristic

The mean ± SD and median (IQR) Sultan’s scores of patients with a CR were 3.93 ± 0.72 and 4 (4-4), respectively, with the corresponding values in patients with a PR being 2.77 (0.93) and 3 (2-3.5), respectively. The maximum Youden index value was calculated using the Sultan’s score to predict CR using ROC curve analysis. The cut-off value of the Sultan’s score for predicting CR was 3.5, with a sensitivity and specificity of 78.6% and 76.9%, respectively. The area under the curve was 0.827 (95% confidence interval: 0.688-0.966). The ROC curve is illustrated in Figure 3.

## Discussion

**Figure 4:**
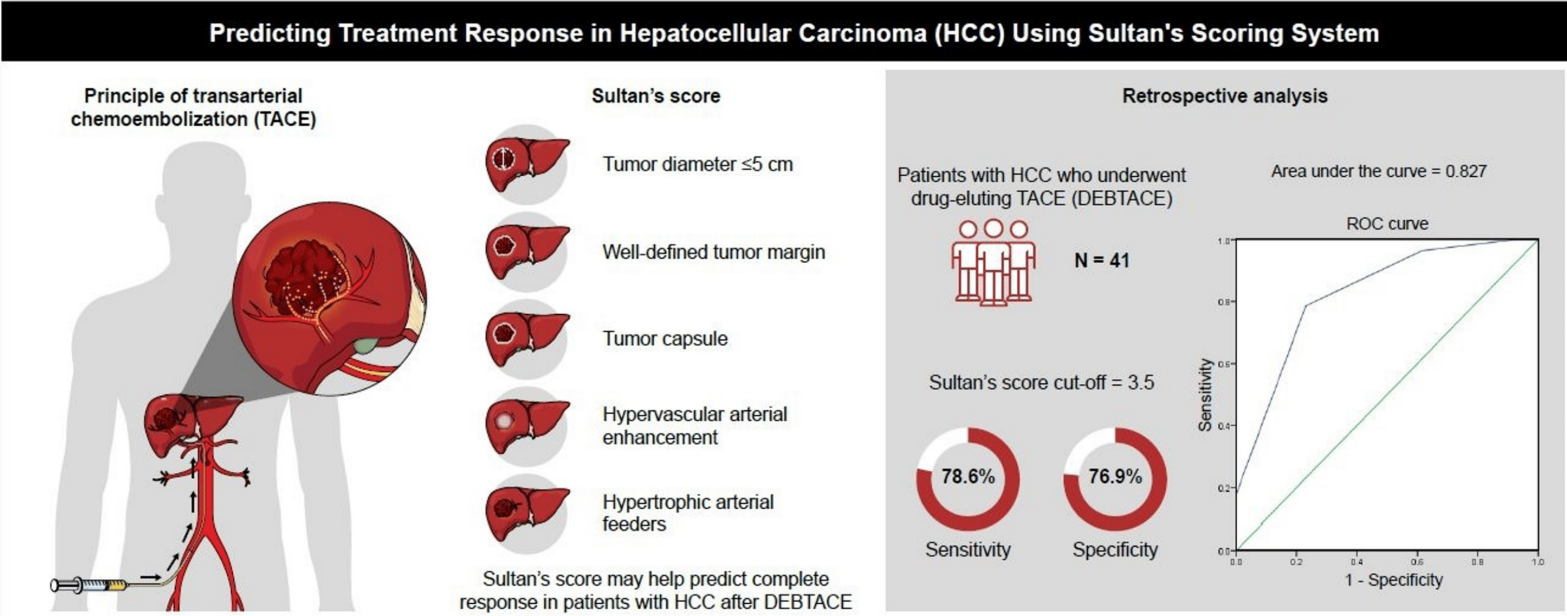
Graphic summary of the new prediction score. (Image created by Sultan Alharbi)

Our study found that the novel imaging-based “Sultan’s Score” accurately predicts the CR of HCC after TACE. Smaller size, well-defined margin, and presence of capsule are significantly associated with CR. Younger patients and Child-Pugh A scores were also found to be associated with CR. The arterial hypervascularity and hypertrophic arterial supply were found to be higher in patients with CR, but statistically non-significant. Most of the treated HCCs were arterially hypervascular tumors, and only a few were hypovascular HCCs, which may lead to statistical non-significance. Our results indicate that arterial hypervascularity and identifying the hypertrophic arterial supply of HCC facilitates embolization of HCC, especially in locations with multiple arterial supplies, such as liver dome, subcapsular HCC, and liver segment 4. While arterial hypervascularity and hypertrophic arterial supply showed higher values in patients with CR, their lack of statistical significance suggests that these features may play a supporting rather than a primary role in predicting response. Figure 4 presents a graphical summary.

Several previous studies have developed scoring systems for predicting survival and treatment response of TACE in patients with HCC [13,14,15]. These models have been based on multiple parameters, including tumor number, maximum tumor size, liver function, AFP, aspartate transaminase, albumin, and bilirubin levels [13,15]. The hepatoma arterial-embolization prognostic (HAP) scoring system and its modified versions (modified HAP, mHAP2, and mHAP3) are based on the baseline liver function and tumor burden [16]. The Assessment for Retreatment with TACE (ART) scoring system is based on Child-Pugh scores, aspartate transaminase levels, and tumor response [17]. The ABCR scoring system is based on AFP, Barcelona clinic liver cancer, Child-Pugh scores, and response [18]. The ASAR scoring system is based on albumin-bilirubin grade, tumor size, alpha-fetoprotein, and first TACE response [19]. Other scoring systems that utilize the sum of the number and size of HCC tumors, which are termed the six and 12 scores [20], and machine learning algorithms that utilize multiple CT textural analysis algorithms have been used to predict the HCC response and patient survival after TACE [21].

However, none of the previously published prediction scores address the imaging characteristics of HCC apart from the tumor burden described by tumor size and number [13,15,20]. All available scoring systems for TACE have failed in independent external validation, showing only moderate accuracy in individual prognostic predictions [14,22]. The receiver operating characteristic (ROC) curve analysis showed that none of the scores had an area under the curve (AUC) over 70% [23]. The AUC of HAP, ART, ABCR, ASAR, and the six- and 12-score systems are 0.63, 0.57, 0.60, 0.59, and 0.67, respectively [13,20,23]. Accordingly, the European Association for the Study of the Liver (EASL) guidelines consider the use of scoring systems controversial and recommend that TACE be stopped after failure to achieve considerable necrosis after two sessions [8].

Several studies have confirmed a correlation between imaging biomarkers and TACE efficacy [8,9]. The best therapeutic effect of TACE is seen in simple encapsulated nodular HCCs, whereas infiltrative HCCs are characterized by a high frequency of vascular invasion and poor treatment response [24]. The current Barcelona-Clinic Liver Cancer (BCLS) guidelines recommend systemic therapy for intermediate HCC of diffuse and infiltrative HCC since these patients do not benefit from TACE [25]. Small tumor size is a good predictor of tumor response and is considered an independent predictor for achieving a CR; contrastingly, an HCC size ≥5 cm is associated with a CR rate of <20%, and an HCC size >10 cm is generally considered a contraindication for TACE [20,26,27].

In contrast to previously reported TACE response prediction scoring systems, this novel score exhibited a higher sensitivity (78.6%), specificity (76.9%), and area under the curve of (0.827, 95% CI: 0.688-0.966) in predicting CR after two sessions of DEBTACE. It is an easy and readily available score that is based on the imaging features of HCC. Each of the HCC imaging features, including well-defined borders, encapsulation, hypervascularity, smaller tumor size, and presence of hypertrophic arterial feeders, is well known to have a better response after TACE. The score is the summation of the five features, with the highest score indicating a better prediction of complete response post-TACE.

The potential clinical implications of this novel score on HCC treatment are significant. Patients with higher scores are expected to achieve a complete radiological response making them ideal candidates for DEBTACE with curative intent. On the other hand, patients with low scores are less likely to experience a complete radiological response. Therefore, alternative treatment or a combination of TACE with other therapies would be more suitable for these patients. 

Notably, this study had several limitations. First, retrospective study designs have an inherent bias. Second, the study included patients with solitary HCC. Third, the study has a single-center setting with a small sample size. Fourth, it lacks external validation. Despite these limitations, our score seems helpful in predicting the HCC complete response after TACE. 

## Conclusions

Patients with HCC responses to TACE vary greatly. We developed a novel predictive score (Sultan’s score) to predict a complete response after TACE treatment. The Sultan’s score comprises five elements based on imaging features, including well-defined borders, tumor capsule, size, arterial hyperenhancement, and hypertrophic arterial feeder. The Sultan’s score can help in predicting patient responses. However, external validation and prospective studies with larger sample sizes and multifocal HCC patients are required to validate these findings.
